# Greater utility of molecular subtype rather than epithelial‐to‐mesenchymal transition (EMT) markers for prognosis in high‐risk non‐muscle‐invasive (HGT1) bladder cancer

**DOI:** 10.1002/cjp2.167

**Published:** 2020-05-06

**Authors:** Edward C Ottley, Robert Pell, Benedict Brazier, Julianne Hollidge, Christiana Kartsonaki, Lisa Browning, Eric O'Neill, Anne E Kiltie

**Affiliations:** ^1^ CRUK/MRC Oxford Institute for Radiation Oncology University of Oxford Oxford UK; ^2^ Nuffield Department of Surgical Sciences University of Oxford Oxford UK; ^3^ Department of Population Health University of Oxford Oxford UK; ^4^ Department of Cellular Pathology, and the NIHR Oxford Biomedical Research Centre John Radcliffe Hospital Oxford UK

**Keywords:** bladder cancer, biomarkers, epithelial‐to‐mesenchymal transition, immunohistochemistry, molecular subtypes

## Abstract

Approximately 75% of bladder cancers are non‐muscle invasive (NMIBC). Of these, up to 53% of cases progress to life‐threatening muscle‐invasive bladder cancer (MIBC). Patients with high‐grade stage T1 (HGT1) NMIBC frequently undergo radical cystectomy (RC), although this represents overtreatment for many. Identification of progressors versus non‐progressors could spare unnecessary treatment. Recent studies have confirmed that urothelial carcinoma is composed of two main molecular subtypes, basal and luminal, with 12% of basal tumours exhibiting epithelial‐to‐mesenchymal transition (EMT). Levels of immune cell infiltration have been shown to be subtype‐specific. Here, we performed immunohistochemistry (IHC) for 11 antibodies relating to molecular subtypes or EMT in 26 cases of HGT1 urothelial carcinoma cases with 6 matched samples subsequently obtained at cystectomy (*n* = 6; 1 muscle‐invasive, 5 non‐muscle‐invasive; 3 = pTis, 1 = pT1, 1 = pTa) and at recurrence (*n* = 2, pT2). RNAScope was also conducted in a subset. Expression patterns in HGT1 specimens versus MIBC (pT2+) were examined, and correlated with disease‐specific survival (DSS). Levels of stromal tumour‐infiltrating lymphocytes (sTILs) were assessed manually to determine whether lymphocyte infiltration was associated with DSS and whether differences existed between HGT1 and MIBC. Molecular subtype markers demonstrated increased prognostic potential compared to the EMT markers assessed. Increased expression of the luminal markers FOXA1 and *SCUBE2*, were found to be significantly associated with better DFS. No EMT markers were significantly associated with DFS. In areas of non‐invasive papillary urothelial carcinoma, but not invasive carcinoma, sTIL levels were found to be significantly associated with DFS. While differences were observed between HGT1 cases that progressed versus those that did not, a larger cohort study is required for validation of these findings. Taken together, an emphasis on molecular subtype markers, rather than EMT markers, may be preferable when studying biomarkers of HGT1 urothelial carcinoma in the future.

## Introduction

Approximately 75% of newly diagnosed bladder cancers are non‐muscle invasive (NMIBC), rather than muscle‐invasive (MIBC) [1], with 25% of cases showing lamina propria invasion. Up to 53% of these cases can progress from NMIBC (pTa or pT1) to life‐threatening muscle‐invasive bladder cancer (MIBC, pT2+) [2]. Patients with high‐grade stage T1 (HGT1) NMIBC may undergo radical cystectomy (RC), although this represents overtreatment in many patients [2]. There is therefore an urgent clinical need to identify likely progressors versus non‐progressors, for patients to be given appropriate treatment promptly or to be spared unnecessary treatment.

Cancer cells undergoing epithelial‐to‐mesenchymal transition (EMT), a mechanism of invasion, are characterised by a loss of proteins that mediate cell–cell contacts (e.g. E‐cadherin) and increased expression of markers that promote a motile cell state (vimentin and N‐cadherin) [3]. In some cases, EMT is a reversible process, with mesenchymal‐to‐epithelial transition (MET) also possible. This is observed in normal embryonic development but has also been demonstrated in cancer cells *in vitro* and in mouse xenografts [3, 4]. The reversible nature of EMT makes it an attractive therapeutic target. Indeed, factors relating to EMT have been shown to be druggable targets [5, 6]. Notably, monoclonal antibody (mAb) and small molecule targeting of receptor tyrosine kinases (RTKs), activation of which can promote EMT, have either been approved or are under clinical trial [6]. If EMT could be prevented, halted or reversed in patients identified as being at risk of progression to MIBC, this could obviate the need for cystectomy. While EMT has been associated with bladder cancer progression and metastasis at the genomic level [7], the patterns of EMT markers at the protein level and the contribution to HGT1 disease are not well understood.

A range of studies have investigated molecular subclassification of bladder and recently a six‐class consensus classification has been reached [8, 9, 10, 11, 12]. Although each classification varies slightly, with Sjodahl *et al* [8] also evaluating NMIBC, two major subtypes, basal and luminal, have emerged with further subclassifications within them. Approximately 12% of tumours are claudin‐low (a basal subtype) and exhibit EMT; in contrast, luminal tumours (66% of cases) maintain E‐cadherin expression, demonstrating a link between molecular subtype and EMT [10]. Ultimately, despite the large number of independent studies investigating the molecular subtypes of bladder cancer, it is the two main subtypes, basal and luminal, that are common to all studies [13]. However, these are not currently used in clinical practice.

In the latest TCGA molecular subtype study, one of the luminal subtypes, luminal papillary (LumP), was found to have a low risk for metastasis [14]. This implies that for HGT1 disease, the molecular subtype of the tumour may contribute to risk of progression to MIBC. Different oncogenic mechanisms are also present between the different consensus molecular subtypes. For instance, LumP tumours are strongly characterised by increased FGFR3 transcriptional activity as opposed to basal/squamous (Ba/Sq) tumours, which employ EGFR mechanisms [12].

Bladder cancer is highly influenced by the immune system. This is particularly reflected in the fact that administration of Bacillus–Calmette–Guerin (BCG) has been used since the 1970s as a proven effective treatment of high‐grade NMIBC to prevent progression to MIBC [15]. This stimulation of the immune system therefore has the potential, in some cases, to act as a ‘brake’ to progression. The importance of immune characteristics, particularly with respect to immune infiltration, is reflected in the latest MIBC molecular subtypes consensus [12]. Differences in the overall level and components of infiltration was dependent on subtype. For example, the LumP, luminal unstable and neuroendocrine‐like subtypes all exhibited low levels of immune infiltration (‘immune‐cold’) compared to the luminal non‐specified, stroma‐rich and Ba/Sq. These differences between subtypes also have implications for therapy, with some Ba/Sq tumours likely to respond well to immunotherapies, while other tumours, such as LumP, are likely to benefit from other forms of treatment.

Tumour infiltrating lymphocytes (TILs) are important in a range of different cancers and, generally, increased TILs are associated with better prognosis [16, 17, 18, 19]. Numerous groups have investigated intratumoural, intra‐epithelial and stromal TILs (sTILs) [20]. sTILs are located between carcinoma cells either in a clustered or dispersed fashion and have no direct contact or interaction with the tumour cells in contrast to TILs. Recent studies have shown sTIL evaluation by pathologists can predict response to treatment with greater consistency than TIL evaluation [20].

The aims of this study were, first, to determine whether an integrated panel of 11 antibodies relating to bladder cancer molecular subtypes or EMT exhibited differential expression patterns in HGT1 specimens versus muscle‐invasive disease, and whether differences were associated with disease‐specific survival (DSS).

Second, we wanted to determine whether differences in molecular subtype‐specific mRNA expression correlated with survival and/or progression to muscle‐invasive disease, using chromogenic RNAScope *in situ* hybridisation, and whether lymphocyte infiltration played a crucial role in this. This study was a pilot study involving a small number of cases but a relatively large panel of antibodies, with a view to identifying a smaller panel of antibodies to study in a larger cohort.

We hypothesised that HGT1 tumours with a propensity for EMT, high basal marker expression (at protein and mRNA level) and high sTIL expression would be more likely to progress to MIBC, with a poorer prognosis.

## Materials and methods

### Tissue information

#### Immunohistochemistry

Patients with HGT1 urothelial carcinoma of the bladder diagnosed between 2009 and 2015 were identified in the Oxford Radcliffe Biobank (ORB; *n* = 26). Under ethical approval (South Central Oxford REC C 09/H0606/5+5), 4 μm‐thick sections were taken from formalin‐fixed paraffin‐embedded (FFPE) tissue obtained at transurethral resection (TURBT) from these patients. While for most patients this was their first presentation with bladder cancer, two were noted to have had previous HGT1 disease on their pathology reports (one 7 years previously who subsequently had a cystoprostatectomy) and one to have had previous low grade NMIBC. Sections were also taken from samples in patients whose tumour recurred (T2 samples at recurrence [*n* = 2] or obtained at cystectomy [*n* = 6; 1 muscle‐invasive, 5 NMIBC; 3 = pTis, 1 = pT1, 1 = pTa]). Corresponding H&E stained sections were assessed by a consultant histopathologist (LB) and normal urothelium, carcinoma *in situ* (CIS), papillary tumour and invasive tumour regions (T1 or T2), avoiding diathermied areas and crush artefact, were outlined to aid subsequent analysis.

#### RNAScope

A subset of samples was used for this assay, including HGT1 (*n* = 13) and recurrence/cystectomy cases (*n* = 4).

### Immunohistochemistry

Automated immunohistochemistry (IHC) was conducted on the sections using a Bond‐max autostainer (Leica Microsystems, GmbH, Wetzlar, Germany). Sections were incubated with primary antibodies at the dilutions and incubation times described in Supplementary material, Table S1. Stained slides were digitised using an Aperio ScanScope CS2 digital slide scanner (Leica Microsystems) at ×400 total magnification. Positive and negative controls were included in all runs (see Supplementary material, Figure S1). See supplementary material, Materials and methods for additional information.

### RNAScope *in situ* hybridisation

RNAScope *in situ* hybridisation (ISH) was performed on FFPE tissue sections (6 μm) as per the manufacturer's guidelines (Advanced Cell Diagnostics, Hayward, CA, USA). Hybridisation probes used were *FGFR3*, *EGFR*, *SCUBE2* and *ZEB2*. See Supplementary material, Table S2 for further details. Probes were added to each tissue section and allowed to incubate for 2 h at 40°C. For detection, the RNAScope 2.5 Detection Reagent—Red kit was used (Cat#322360; Advanced Cell Diagnostics, Hayward, CA, USA). Positive control sections detected the presence of human peptidylprolyl isomerase B (hs‐PPIB) and DapB in negative control sections; these were included in each assay (representative images in Supplementary material, Figure S2). See supplementary material, Materials and methods for additional information.

### IHC and ISH assessment

#### Antibodies

Antibodies used are shown in Supplementary material, Table S1. To assess EMT, N/E‐cadherin, Axl, vimentin and slug/snail antibodies were used. For the molecular subtypes, basal markers included CK5/6, CK14 and CD44; luminal markers were GATA3, FOXA1 and CK20. See supplementary material, Materials and methods for further details.

#### Immunohistochemistry assessment

For Cytokeratins 5/6, 14 and 20, CD44, vimentin and Axl, cases were classified as negative or positive based on criteria from the literature (by ECO; Table 1). For FOXA1, GATA3, E‐cadherin, N‐cadherin and slug/snail, staining was assessed using a semi‐quantitative H‐score method in papillary (*n* = 24–28), invasive (*n* = 14–16), normal (*n* = 5–8) and CIS (*n* = 3–5) regions (by ECO) [21]. See supplementary material, Materials and methods, and Figure S3 for more information.

**Table 1:** Scoring methods used for antibodies.

Antibody	Positive classification	Negative classification	Reference
Basal			
CK‐5/6	Moderate to strong staining	Normal staining in basal/parabasal cells. Absent staining	Desai *et al* [41]
CK‐14	Moderate to strong staining	Normal staining in basal/parabasal cells. Absent staining	Desai *et al* [41]
CD44	Positive staining throughout the tumour cells	Normal staining in basal/parabasal cells, ‘patchy‐loss of staining’ or absent staining	Desai *et al* [41]
Luminal			
CK‐20	≤10% focal positive staining or ≥10% diffuse positive staining	Normal positive staining of superficial umbrella cells **or** absent staining	Desai *et al* [41]
GATA3	H‐score method – nuclear		Walker *et al* [21]
FOXA1	H‐score method – nuclear		Walker *et al* [21]
EMT			
Vimentin	≥5% tumour cytoplasmic positive staining	≤5% tumour cytoplasmic positive staining	Sanfrancesco *et al* [42]
E‐cadherin	H‐score method – membranous		Walker *et al* [21]
N‐cadherin	H‐score method – membranous		Walker *et al* [21]
Slug/snail	H‐score method – nuclear		Walker *et al* [21]
Axl	≥5% positive membranous and cytoplasmic staining	≤5% positive membranous and cytoplasmic staining	Shieh *et al* [43]

Positive and negative criteria were established for relevant stains using information from the literature. H‐score was determined as the product of percentage of positive cells and staining intensity (graded 0, 1+, 2+, 3+), as previously described [21].

#### RNAScope *in situ* hybridisation quantification

To quantify the RNAScope staining, HALO software with an accompanying ISH algorithm was utilised. Slides were digitalised using an Aperio CS2 bright field scanner (Leica Biosystems, San Diego, CA, USA) and a total of 10 images per region (papillary [*n* = 12], invasive [*n* = 13], CIS [*n* = 2] and normal urothelium [*n* = 3]) per slide were extracted for subsequent assessment. The ISH algorithm was used to determine average probe area per μm^2^ for each image for each region, as previously described [22]. Mean probe area per μm^2^ was determined for all 10 images for each region present on each slide. Data was analysed further both in a regional and globally dependent manner. For global assessment, ‘case score’ was calculated by generating an average probe area per μm^2^ for all regions assessed for a particular case.

### Manual sTIL assessment

Quantification of percentage coverage of stroma adjacent to the papillary and invasive regions of tumour on Digital Images of H&E‐stained tissue sections was undertaken following the technique described by Denkert *et al* [20, 23] using an open source Digital Software Platform (QuPath) [24] by a single pathologist observer (RP). Regions of focal invasive disease contained within papillary cores were excluded from assessment. The sections were scored in triplicate and the mean score for each region was used for analysis. The investigating pathologist was blinded to the details and outcomes of each case. In addition, as no formal recommendation for a clinically relevant threshold has been previously published, sTIL data was analysed as a linear parameter.

### Statistical analysis

To determine whether any differences existed between normal, CIS and invasive regions, in terms of overall H score, a one‐way analysis of variance (ANOVA) was conducted followed by a Tukey's multiple comparison test. Associations of IHC markers, sTIL and RNAScope with DSS were assessed using Kaplan–Meier curves and Cox proportional hazards models. Individuals who did not die of bladder cancer were censored at last time known to be alive or at time of death from other causes. Time since TURBT was used as the time scale. Markers were used as numeric and also dichotomised. The proportional hazards assumption was assessed by examining scaled Schoenfeld residuals. Correlation coefficients between markers were calculated. Analysis was done using the Statistical Software R [25].

## Results

### Differential staining of basal, luminal and EMT markers in tumour regions

**Figure 1:**
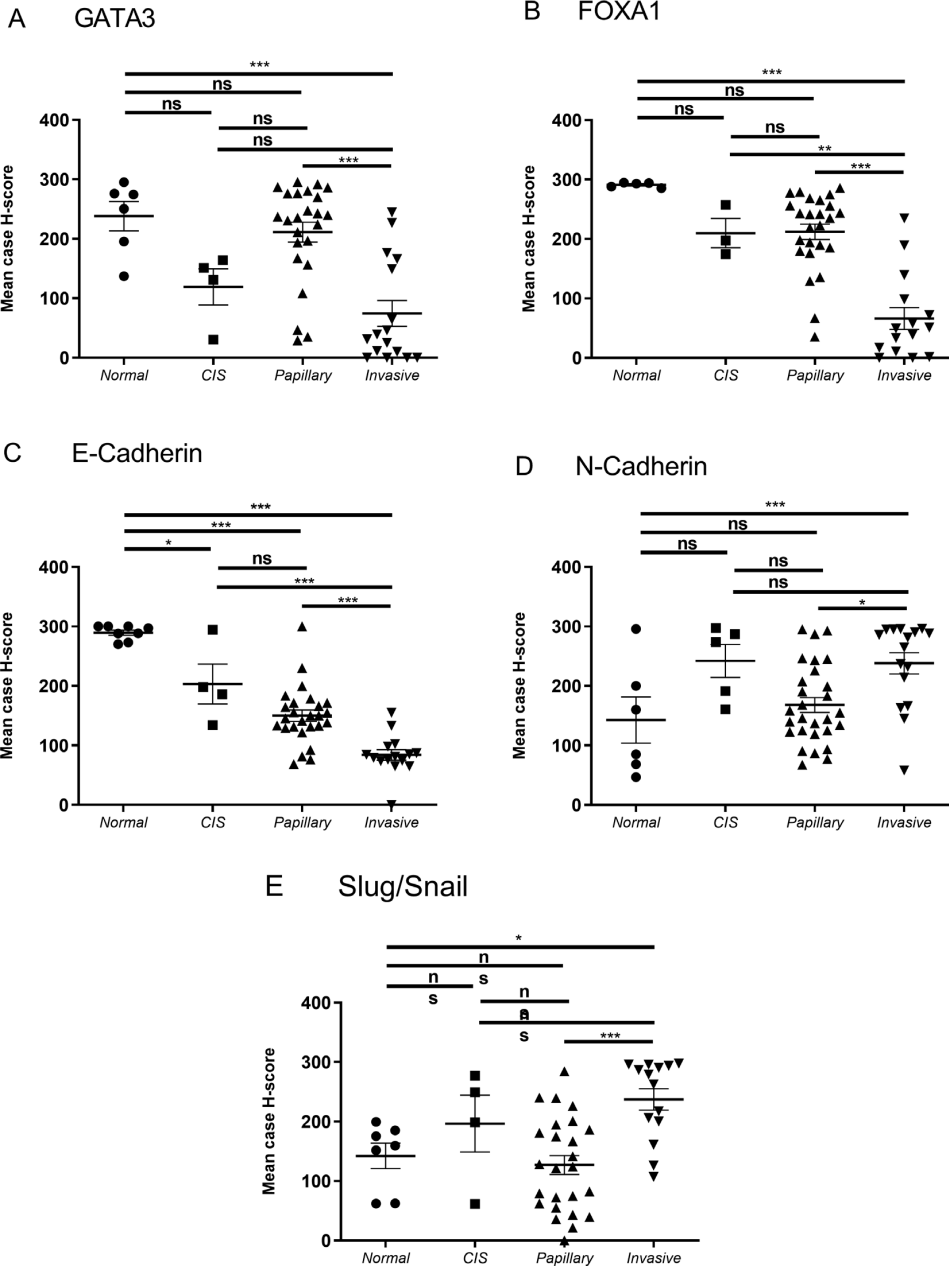
Expression of basal, luminal and EMT markers between tumour regions for antibodies assessed via the H‐score method. The mean case H‐score for normal, CIS, papillary and invasive regions are presented for all cases (HGT1, *n* = 26 and recurrence/cystectomy, *n* = 8). Luminal antibodies (A) GATA3 and (B) FOXA1; and EMT antibodies (C) E‐cadherin, (D) N‐cadherin and (E) slug/snail were assessed. Data are presented as mean case H‐score ± SEM. *p < 0.05, **p < 0.01 and ***p < 0.001 with a one‐way ANOVA followed by Tukey's multiple comparison test (not significant where *p* > 0.05).

We assessed whether there were differences between normal, CIS, papillary and invasive regions of tumour (Figure 1). Using H‐score, the luminal markers FOXA1 and GATA3 showed a similar trend. A significant difference in expression was observed between normal and invasive areas and papillary and invasive for both GATA3 and FOXA1. In addition, a significant decrease in FOXA1 expression was observed between CIS and invasive regions. For EMT markers, E‐cadherin expression was lower in invasive areas compared to papillary, CIS and normal regions. N‐cadherin and slug/snail expression was significantly higher in invasive areas, compared to both normal and papillary regions.

Additional basal, luminal and EMT markers were assessed using a positive/negative scoring system. For all basal markers assessed (Table 1), no positive staining was observed in normal and CIS areas and the highest percentage of positive areas was in invasive regions; CD44 positivity was found only in invasive regions (31%). The luminal marker CK20 exhibited the highest positivity (92%) in papillary regions (Table 2).

**Table 2:** The association of regional staining positivity with DSS.

Antibody	Normal	*P* value	CIS	*P* value	Papillary	*P* value	Invasive	*P* value
Molecular subtype markers – assessed with binary (±) method								
CK‐5/6	0	–	0	–	2 (8%)	–	6 (23%)	0.833
CK‐14	0	–	0	–	5 (19%)	0.493	8 (31%)	0.962
CD44	0	–	0	–	0	–	8 (31%)	0.587
CK‐20	1 (4%)	–	3 (12%)	0.809	24 (92%)	–	9 (35%)	0.749
EMT markers – assessed with binary (±) method								
Vim	0	–	0	–	0	–	5 (19%)	0.917
Axl	0	–	0	–	3 (12%)	–	3 (12%)	0.364

*P* values for association with DSS are shown. For instances where *P* values are not present, there were either no positive regions for this antibody, or convergence issues were present due to low numbers.

### Elevated FOXA1 expression was significantly associated with DSS

Of all of the antibodies assessed via the H‐score method, papillary FOXA1, per unit increase, was significantly associated with DSS in terms of % positive (*p* = 0.02), intensity (*p* = 0.035) and overall H‐score (*p* = 0.019; Table 3). For antibodies assessed using the positive/negative method, no antibodies were found to be significantly associated with DSS.

**Table 3:** Association with DSS for EMT and molecular subtype antibodies assessed with the H‐score method.

Antibody and region	logHR	HR	se(logHR)	*P* value	95% CI
GATA3 papillary % positive	−0.015	0.985	0.014	0.278	(0.958, 1.012)
GATA3 papillary intensity	−0.564	0.569	0.379	0.136	(0.271, 1.195)
GATA3 papillary H‐score	−0.004	0.996	0.004	0.251	(0.988, 1.003)
GATA3 invasive % positive	−0.007	0.993	0.013	0.588	(0.968, 1.019)
GATA3 invasive intensity	−0.123	0.884	0.48	0.798	(0.345, 2.266)
GATA3 invasive H‐score	−0.002	0.998	0.005	0.741	(0.989, 1.008)
**FOXA1 papillary % positive**	−0.05	0.952	0.021	**0.020**	(0.913, 0.992)
**FOXA1 papillary intensity**	−1.228	0.293	0.581	**0.035**	(0.094, 0.914)
**FOXA1 papillary H‐score**	−0.012	0.988	0.005	**0.019**	(0.978, 0.998)
FOXA1 invasive % positive	−0.044	0.957	0.024	0.066	(0.913, 1.003)
FOXA1 invasive intensity	−0.877	0.416	0.584	0.133	(0.133, 1.306)
FOXA1 invasive H‐score	−0.015	0.985	0.01	0.139	(0.965, 1.005)
E‐Cad papillary % positive	0.015	1.015	0.038	0.691	(0.943, 1.093)
E‐Cad papillary H‐score	0.018	1.018	0.013	0.154	(0.993, 1.044)
E‐Cad invasive % positive	−0.032	0.969	0.023	0.163	(0.926, 1.013)
E‐Cad invasive intensity	−0.377	0.686	2.157	0.861	(0.01, 47.042)
E‐Cad invasive H‐score	0.007	1.007	0.02	0.735	(0.967, 1.048)
N‐Cad papillary % positive	0	1	0.035	0.992	(0.934, 1.07)
N‐Cad papillary intensity	−0.307	0.735	0.552	0.578	(0.25, 2.168)
N‐Cad papillary H‐score	−0.002	0.998	0.005	0.703	(0.987, 1.009)
N‐Cad invasive % positive	−0.002	0.998	0.04	0.954	(0.923, 1.078)
N‐Cad invasive intensity	−0.912	0.402	1.226	0.457	(0.036, 4.439)
N‐Cad invasive H‐score	−0.004	0.996	0.009	0.671	(0.978, 1.015)
Slug/snail papillary % positive	−0.029	0.972	0.02	0.149	(0.935, 1.01)
Slug/snail papillary intensity	−0.634	0.53	0.399	0.112	(0.242, 1.16)
Slug/snail papillary H‐score	−0.007	0.993	0.005	0.16	(0.983, 1.003)
Slug/snail invasive % positive	−0.028	0.973	0.033	0.393	(0.913, 1.037)
Slug/snail invasive intensity	0.259	1.296	1.108	0.815	(0.148, 11.37)
Slug/snail invasive H‐score	−0.001	0.999	0.008	0.898	(0.984, 1.014)

Significant *P* values (*p* < 0.05) are in bold.

### Differences in sTIL percentages between tumour regions and association with DSS

**Figure 2:**
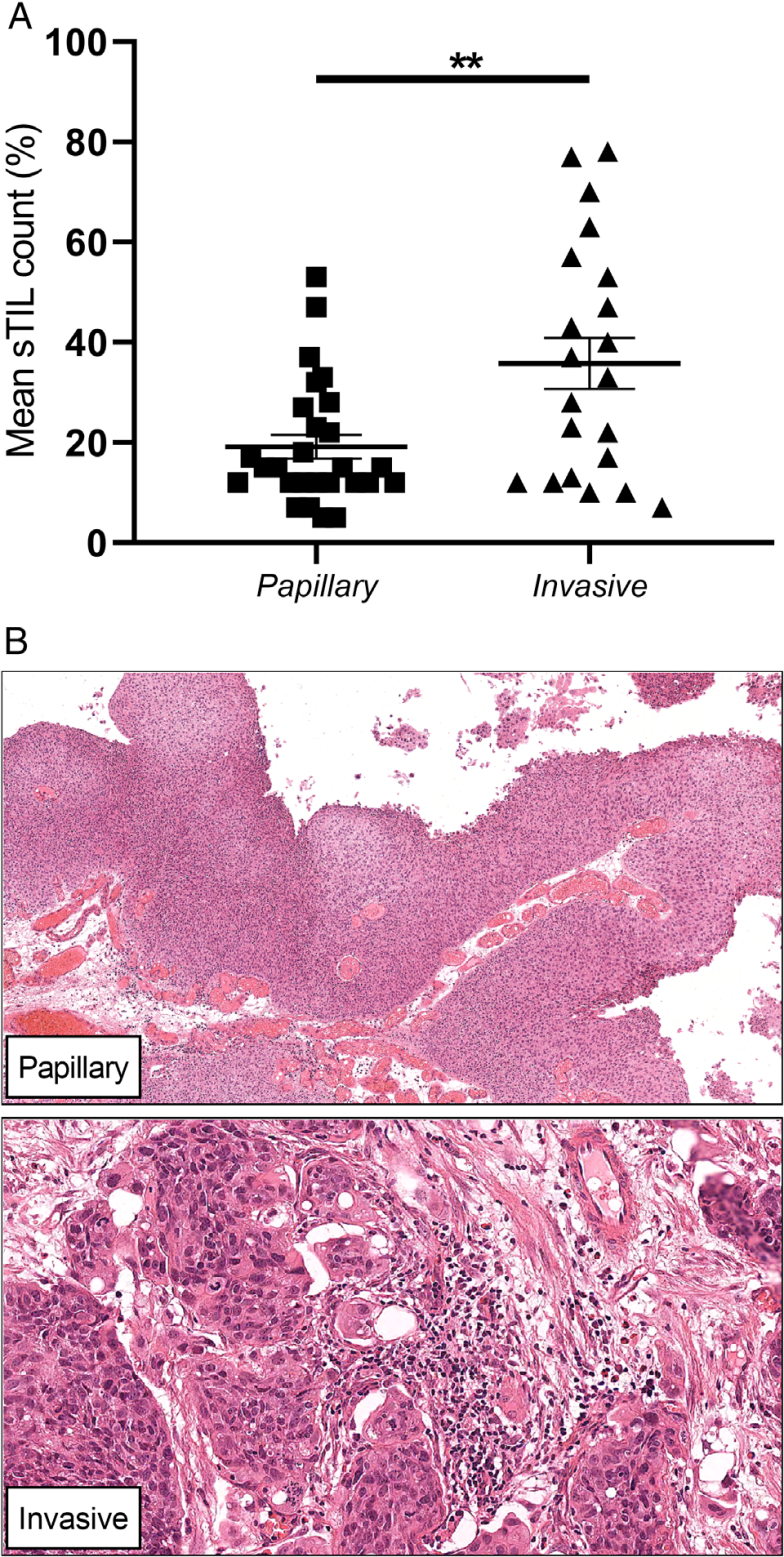
Regional sTIL expression data. (A) Differences between papillary and invasive tumour regions. Data presented as mean sTIL count ± SEM. **p < 0.01 by unpaired Student's *t*‐test. (B) Representative images for each region assessed.

Significant increases in sTIL levels were seen between papillary and invasive regions (*p* < 0.01; Figure 2A), and see representative images for each region (Figure 2B). Papillary sTIL levels were significantly associated with DSS (*p* < 0.05; Table 4).

**Table 4:** Association of papillary and invasive sTIL% on DSS.

Tumour region	logHR	HR	se(logHR)	*P* value	95% CI
Papillary	0.056	1.057	0.028	**0.043**	(1.002, 1.116)
Invasive	0.023	1.023	0.015	0.123	(0.994, 1.054)

The significant *P* value (*p* < 0.05) is in bold.

The association with DSS was calculated for sTILs quantified from papillary and invasive regions. The logHR, HR, se(logHR), *P* value and 95% CI are presented.

### RNAScope ISH probe expression patterns in invasive and papillary regions

**Figure 3:**
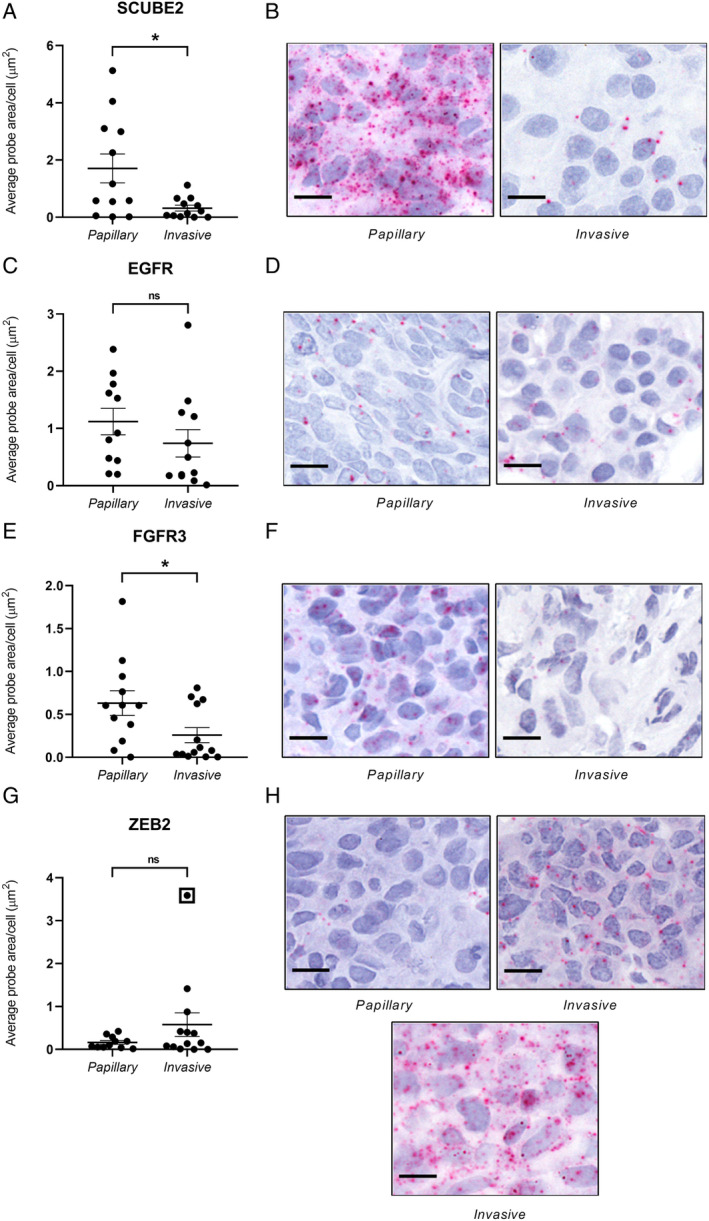
Regional RNAScope probe expression. Papillary and invasive region expression for RNAScope probes (A, B) *SCUBE2*, (C, D) *EGFR*, (E, F) *FGFR3* and (G, H) *ZEB2* are presented. Representative papillary and invasive images are included for each probe. For *ZEB2*, a case with high expression is shown in the bottom panel of (H). Data are presented as average probe area/cell (μm^2^) ± SEM. *p < 0.05 by unpaired Student's *t*‐test. Scale bars = 10 μm.

RNAScope ISH was used to investigate mRNA expression of *SCUBE2*, *EGFR*, *FGFR3* and *ZEB2* chromogenically in the tissue sections. Marked differences were observed between papillary and invasive regions for a number of probes (Figure 3). Expression of the novel tumour suppressor *SCUBE2* was significantly decreased in invasive regions compared to papillary regions (Figure 3A, *p* < 0.05). Expression of *FGFR3* was also significantly decreased in invasive regions (Figure 3E, *p* < 0.05). No significant differences were seen in *EGFR* (Figure 3C) or *ZEB2* (Figure 3G) expression. However, for *ZEB2*, a number of cases exhibited high expression in invasive regions (Figure 3H).

### RNAScope probe expression and correlation with DSS

**Figure 4:**
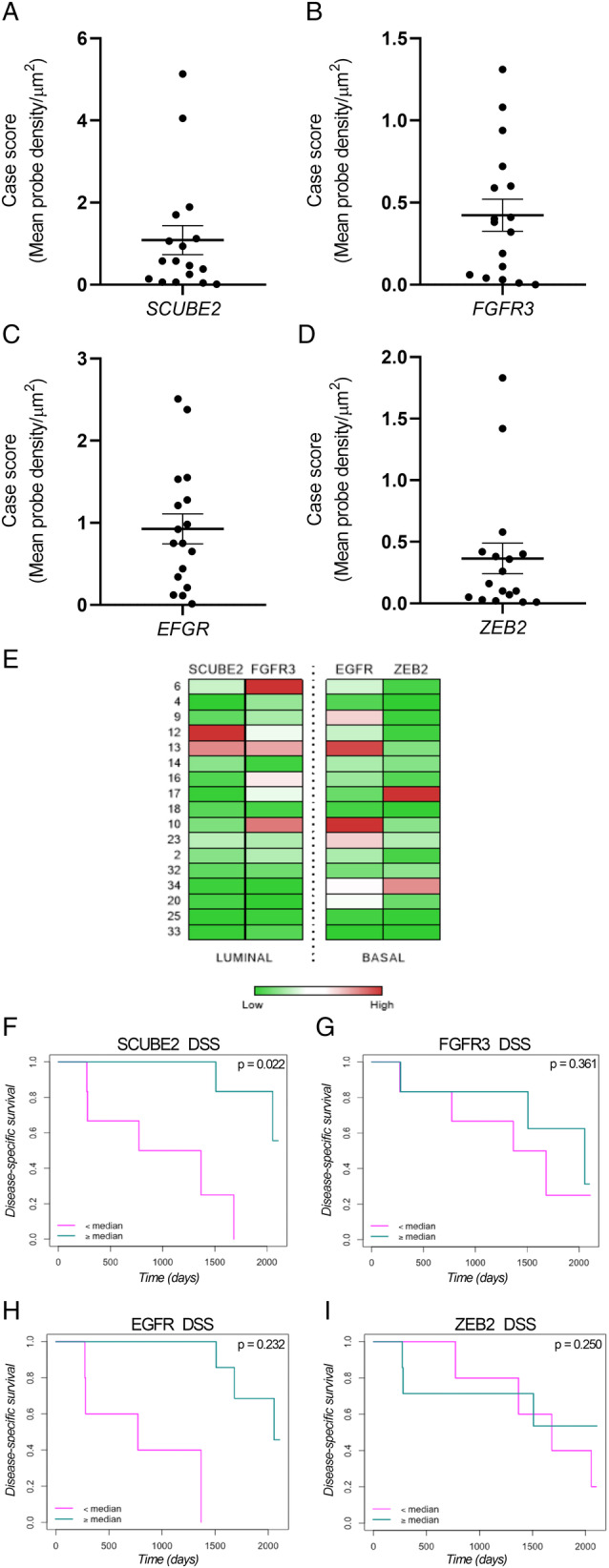
Global RNAScope probe case score and associations with survival. Average probe density/μm^2^ for each case was calculated for each probe (A) *SCUBE2*, (B) *FGFR3*, (C) *EGFR* and (D) *ZEB2*. Data are presented as mean probe density/μm^2^ per case ± SEM. (E) all RNAScope case scores for all cases presented in a heat map format. Kaplan–Meier curves for DSS stratified by median case score for (F) *SCUBE2*, (G) *FGFR3*, (H) *EGFR* and (I) *ZEB2*.

For each case, the mean case score was calculated (Figure 4A–D). Case scores for each probe were visualised as a heat map (Figure 4E), stratified at the median value and correlated with DSS (Figure 4F–I). *SCUBE2* was significantly associated with DSS when stratified at the median (*p* = 0.022). DSS for regional RNAScope probe expression is shown in Supplementary material, Figure S4.

### Integration of staining patterns and comparison of HGT1 cases with their respective cystectomy samples

**Figure 5:**
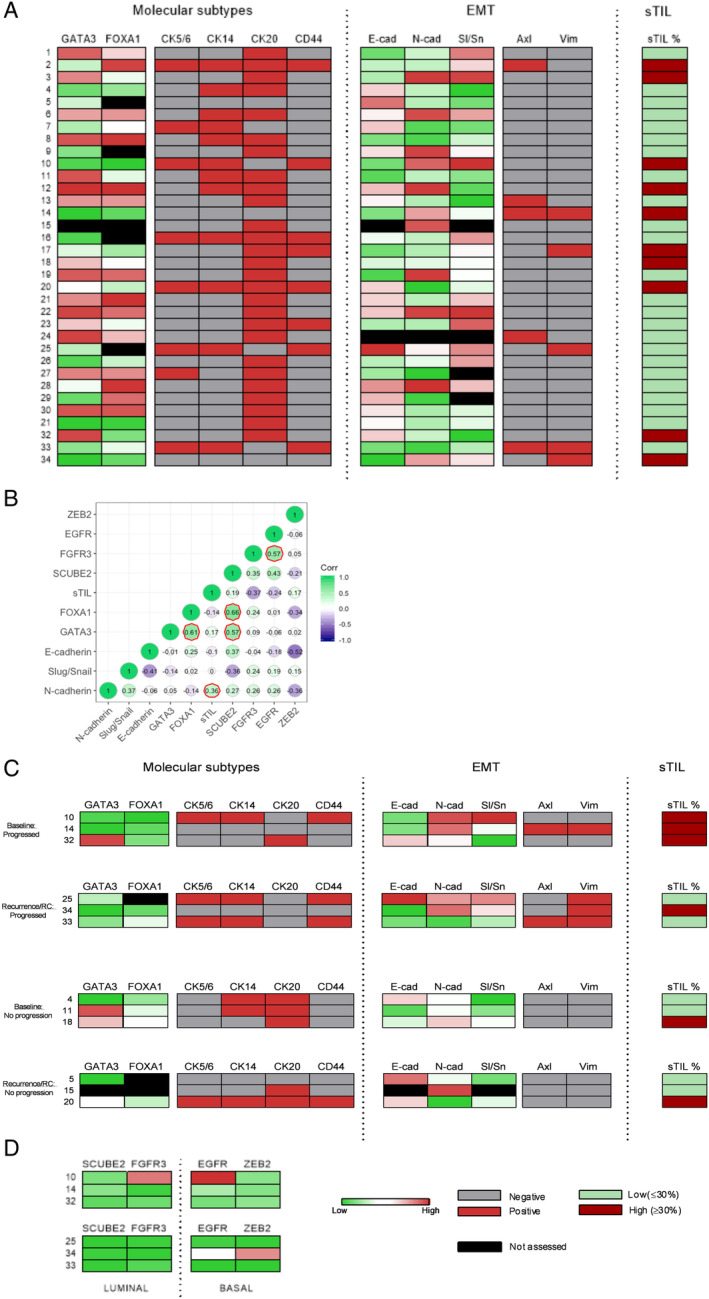
Heat maps for IHC and ISH expression and sTIL percentage. (A) IHC for molecular subtype and EMT markers and sTIL percentages for all cases (HGT1 and recurrence/cystectomy). (B) A correlation matrix for all H‐score, ISH and sTIL data. Significant correlations are outlined in red (*p* < 0.05), with a Pearson's product–moment correlation test. (C) Cases were then sub‐divided into baseline HGT1 cases that progressed and recurrence/RC cases that progressed. Cases were also sub‐divided into heat maps for HGT1 cases that did not progress and recurrence/RC cases that did not progress. (D) RNAScope expression data for baseline HGT1 cases that did progress and recurrence/RC cases that progressed.

We consolidated all IHC, ISH and sTIL data into expression heat maps and a correlation matrix was generated (Figure 5A–D). For basal and luminal staining, a range of patterns was observed. Three cases (cases 2, 16 and 20) were positive for all basal and luminal markers assessed. Most cases were positive for CK20. In cases that were negative for CK20 (cases 5, 7, 10, 14, 25, 33 and 34) four demonstrated high N‐cadherin and/or slug/snail expression (cases 10, 14, 25 and 34) and four cases (cases 14, 25, 33 and 3) demonstrated positivity for either Axl or vimentin or both. In relation to the EMT markers assessed, inverse expression was mostly present between E‐cadherin and N‐cadherin/slug/snail. High expression of N‐cadherin was regularly associated with high nuclear slug/snail expression. In three of five cases positive for vimentin, positive staining for at least one basal marker was observed (cases 17, 25 and 33) with some cases positive for all basal markers assessed (cases 25 and 33). For all H‐score IHC data, ISH case scores and sTIL data, a correlation matrix was constructed (Figure 5B). Significant negative correlations were observed for slug/snail and E‐cadherin (*p* < 0.05) and E‐cadherin and *ZEB2* (*p* < 0.05). Positive correlation was observed for GATA3 and FOXA1 (*p* < 0.01).

For select HGT1 cases, where matched cystectomy or recurrence samples were available we looked for differences in patients who progressed beyond pT1 versus those who did not (Figure 5C). A total of three cases with matched cystectomy/recurrence specimens progressed to muscle‐invasive disease (cases 10, 14 and 32). N‐cadherin expression was higher at baseline in cases that progressed (cases 10, 14 and 32) versus those that did not (cases 4, 11 and 18), with high expression maintained in two of three cases to cystectomy/recurrence (cases 25 and 34). In baseline HGT1 cases that progressed, only one of three cases was positive (case 14), however, upon recurrence/cystectomy, all cases were positive for vimentin (cases 25, 33 and 34). In contrast, all cases that did not progress were negative for Axl and vimentin at baseline (cases 4, 11 and 18) and recurrence/cystectomy (cases 5, 15 and 20). In two of three cases that progressed, moderate to high expression of slug/snail was evident compared to cases that did not progress where three cases exhibited low expression of slug/snail (cases 4, 11 and 18). For cases that did not progress, expression of both Axl and vimentin at baseline and recurrence/RC was negative for all cases.

GATA3 expression was relatively high in two of three baseline cases that did not progress (cases 11 and 18) versus those that did (cases 10 and 14). CK20 positivity was also observed in all of these cases, while all cases were negative for CK5/6 and CD44. For those that did progress, CK20 was negative in all recurrence/cystectomy cases.

All cases that progressed to muscle‐invasive disease exhibited high sTIL infiltration levels and these were associated with moderate to high expression of N‐cadherin and/or slug/snail.

For RNAScope probe expression, for baseline cases that progressed (Figure 5D), one out of three cases exhibited high expression of the basal probe *EGFR*. For recurrence/RC cases that progressed luminal probes *SCUBE2* and *FGFR3* were low for all cases, while basal markers *EGFR* and *ZEB2* were increased in case 34.

## Discussion

The aims of this study were to determine whether an integrated panel of 11 antibodies relating to bladder cancer molecular subtypes or EMT exhibited differential expression patterns in HGT1 specimens versus muscle‐invasive disease, and whether differences were associated with DSS. The intention was to ascertain which of the 11 antibodies would be worthy of further validation in a larger cohort, with a view to determining their potential clinical utility. Second, we wanted to determine whether differences in molecular subtype‐specific mRNA expression correlated with survival and/or progression to muscle‐invasive disease. Lymphocyte infiltration levels were also investigated. We hypothesised that high grade tumours with a combined EMT and basal phenotype with low sTIL density would be more likely to progress to MIBC and demonstrate inferior DSS.

The markers evaluated lacked association with progression to muscle‐invasive disease but our study was underpowered (*n* = 3). Assessment of larger cohorts is required to determine whether EMT and molecular subtypes play a role in progression to MIBC. The study was constrained by available patients with matching tissue in a single large centre.

However, interesting observations were found in relation to DSS in a number of the markers that were assessed. Luminal markers FOXA1 and *SCUBE2* were both significantly associated with DSS. Having been described previously to play a role in bladder cancer, in terms of the ability to drive a luminal subtype [9, 14, 26], FOXA1 has also been shown to be involved in other cancers, particularly breast cancer, where it has been shown to be a prognostic factor [27, 28].

In an IHC study for 16 markers which included molecular subtype (FOXA1, GATA, CK14, FGFR3 and EGFR) and EMT (ZEB2) in 519 patients who underwent RC, DSS was not affected by a single marker, except in the case of FOXA1 [29]. These authors' findings for FOXA1 and DSS are supportive of ours, and ZEB2 was found not to be significant.

Given the molecular subtype‐specific nature and previously described prognostic potential of lymphocyte infiltration in a range of tumours [12, 16, 17, 18, 19], we assessed sTIL levels in the HGT1 cohort. Stromal lymphocyte infiltration has been previously assessed in HGT1 bladder cancer but failed to demonstrate prognostic utility [30]. Our findings, in relation to invasive tumour regions, support that study. However, in our study, increased immune cell infiltration around the base of papillary *in situ* regions was significantly associated with lower DSS. When tumours have an intact basement membrane, evasion of cytotoxic T cell response from the stroma is less likely. A rational hypothesis explaining why lymphocytes are aggregating around the base of non‐invasive papillary areas of tumour could be the presence of microinvasion. This has previously been observed in both flat CIS and non‐invasive areas of papillary carcinoma [31]. The areas of microinvasion might be difficult to discern depending on the plane and/or orientation of the section being examined, and microinvasion can be overlooked if there are only a few invasive cells [31, 32]. An alternative hypothesis is that these tumours could have acquired the capability to evade host cytotoxic T cell responses prior to the development of invasive disease, which could lead to localised aggregations of lymphocytes. In this case, the histological response pattern would subsequently persist following the development of invasive disease, which occurs in the context of an established immuno‐privileged state [33]. Tumours with this particular phenotype are therefore, in theory, more likely to progress to muscle‐invasive and disseminated disease due to a dampened host immunological response. Notably, sTIL levels for all baseline HGT1 cases that progressed to muscle‐invasive disease were high. Comparative analysis of regional immune cell infiltrates and associated transcriptomic and protein expression will be required to investigate this finding further. It is also plausible that regional immune cell infiltrate evaluation could predict response to targeted agents such as pembrolizumab with greater efficacy.

We also demonstrated that *SCUBE2* was significantly associated with DSS. This bladder cancer luminal marker [34], is a secreted tumour suppressor protein that has been well characterised in breast cancer [35]. SCUBE2 plays a pivotal role in sonic‐hedgehog (Shh) signalling [36], which is increased in luminal‐papillary, low‐risk, tumours [14]. Taken together, this suggests that increased levels of SCUBE2, and potentially secretion of SCUBE2, could have a protective effect and therefore favour increased DSS However, findings from Islam *et al* (2016) demonstrated that Shh had the potential to promote tumourigenicity via activation of EMT pathways in bladder cancer. Therefore, the exact role of SCUBE2 in bladder cancer, as regards Shh signalling, requires further investigation. Additionally, expression levels of RNA are not always reflected at the protein level. Therefore, in future studies, investigation of SCUBE2 protein expression is required. Given that only four genes were assessed using RNAScope, our results can only be considered preliminary.

A major limitation of this study is that the overall cohort was relatively small. A future multicentre study is required to provide deeper insight into our findings and to build on this pilot study. However, despite the small cohort, FOXA1 was significantly associated with DSS. When taken together with the previously published study, this highlights the potential importance of FOXA1.

Overall, despite multiple EMT markers being evaluated in our study, no clear association with survival could be demonstrated. This is in contrast to the molecular subtype markers and basic spatial localisation of immune infiltrates within the tissue sections, where prognostic association, in papillary regions specifically, was demonstrated. We were initially surprised that none of the EMT markers assessed was associated with survival, having hoped to have elucidated their role in HGT1 disease progression. Other studies had shown associations between EMT marker expression in bladder cancer and survival [37, 38, 39]. Additionally, Otto *et al* [40] demonstrated that aberrant E‐cadherin expression was predictive of T1 progression. However, EMT is just one mechanism of tumour progression. Furthermore, Kollberg *et al* [29] showed that, at the protein level, the only significant association with survival with a univariable analysis was seen for the molecular subtype marker FOXA1 and none of the EMT markers studied. However, it was not significantly associated with survival in a multivariable analysis, indicating that FOXA1 warrants further investigation in future studies.

These initial findings suggest that the identification of poor prognostic groups using standard clinical laboratory techniques might be best achieved by immune infiltrate and molecular subtype evaluation as opposed to EMT markers in HGT1 bladder cancer.

## Author contributions statement

AEK designed the study, supervised the work and revised the manuscript. ECO conducted IHC/ISH staining and related procedures and wrote the manuscript. RP conducted the sTIL assessment and contributed to manuscript preparation. BB assisted with slide digitalisation. JH assisted with patient database retrieval. CK conducted the statistical analyses. LB conducted a pathological assessment of H&E‐stained tissue sections. EO'N contributed to study conception and design. All authors read the final manuscript.

## Supporting information


**Supplementary materials and methods**
**Figure S1.** Positive and negative controls for all antibodies used in IHC
**Figure S2.** RNAScope positive and negative controls
**Figure S3.** H‐score scoring scales and ± examples
**Figure S4.** Disease‐specific survival for regional RNAScope probe expression
**Table S1.** Antibody information
**Table S2.** RNAScope probes

## References

[1] Babjuk M , Burger M , Compérat EM , *et al* European Association of Urology Guidelines on non‐muscle‐invasive bladder cancer (TaT1 and carcinoma *in situ*)‐2019 update. Eur Urol 2019; 76: 639–657. 31443960 10.1016/j.eururo.2019.08.016

[2] Kulkarni GS , Hakenberg OW , Gschwend JE , *et al* An updated critical analysis of the treatment strategy for newly diagnosed high‐grade T1 (previously T1G3) bladder cancer. Eur Urol 2010; 57: 60–70. 19740595 10.1016/j.eururo.2009.08.024

[3] Derynck R , Weinberg RA . EMT and cancer: more than meets the eye. Dev Cell 2019; 49: 313–316. 31063750 10.1016/j.devcel.2019.04.026 PMC7672963

[4] Yoshida T , Ozawa Y , Kimura T , *et al* Eribulin mesilate suppresses experimental metastasis of breast cancer cells by reversing phenotype from epithelial–mesenchymal transition (EMT) to mesenchymal–epithelial transition (MET) states. Br J Cancer 2014; 110: 1497–1505. 24569463 10.1038/bjc.2014.80 PMC3960630

[5] Satelli A , Li S . Vimentin in cancer and its potential as a molecular target for cancer therapy. Cell Mol Life Sci 2011; 68: 3033–3046. 21637948 10.1007/s00018-011-0735-1 PMC3162105

[6] Cho ES , Kang HE , Kim NH , *et al* Therapeutic implications of cancer epithelial‐mesenchymal transition (EMT). Arch Pharm Res 2019; 42: 14–24. 30649699 10.1007/s12272-018-01108-7

[7] Guo CC , Majewski T , Zhang L , *et al* Dysregulation of EMT drives the progression to clinically aggressive sarcomatoid bladder cancer. Cell Rep 2019; 27: 1781–1793. 31067463 10.1016/j.celrep.2019.04.048 PMC6546434

[8] Sjödahl G , Lauss M , Lövgren K , *et al* A molecular taxonomy for urothelial carcinoma. Clin Cancer Res 2012; 18: 3377–3386. 22553347 10.1158/1078-0432.CCR-12-0077-T

[9] Choi W , Porten S , Kim S , *et al* Identification of distinct basal and luminal subtypes of muscle‐invasive bladder cancer with different sensitivities to frontline chemotherapy. Cancer Cell 2014; 25: 152–165. 24525232 10.1016/j.ccr.2014.01.009 PMC4011497

[10] Damrauer JS , Hoadley KA , Chism DD , *et al* Intrinsic subtypes of high‐grade bladder cancer reflect the hallmarks of breast cancer biology. Proc Natl Acad Sci U S A 2014; 111: 3110–3115. 24520177 10.1073/pnas.1318376111 PMC3939870

[11] Network CGAR . Comprehensive molecular characterization of urothelial bladder carcinoma. Nature 2014; 507: 315–322. 24476821 10.1038/nature12965 PMC3962515

[12] Kamoun A , de Reynies A , Allory Y , *et al* A consensus molecular classification of muscle‐invasive bladder cancer. Eur Urol 2020; 77: 420–433. 31563503 10.1016/j.eururo.2019.09.006 PMC7690647

[13] Morera DS , Lahorewala SS , Belew D , *et al* Clinical parameters outperform molecular subtypes for predicting outcome in bladder cancer: results from multiple cohorts including TCGA. J Urol 2020; 203: 62–72. 31112107 10.1097/JU.0000000000000351 PMC8327783

[14] Robertson AG , Kim J , Al‐Ahmadie H , *et al* Comprehensive molecular characterization of muscle‐invasive bladder cancer. Cell 2017; 171: 540–556.e25. 28988769 10.1016/j.cell.2017.09.007 PMC5687509

[15] Meyer J‐P , Persad R , Gillatt DA . Use of bacille Calmette–Guérin in superficial bladder cancer. Postgrad Med J 2002; 78: 449–454. 12185215 10.1136/pmj.78.922.449 PMC1742458

[16] Clemente CG , Mihm MC Jr , Bufalino R , *et al* Prognostic value of tumor infiltrating lymphocytes in the vertical growth phase of primary cutaneous melanoma. Cancer 1996; 77: 1303–1310. 8608507 10.1002/(SICI)1097-0142(19960401)77:7<1303::AID-CNCR12>3.0.CO;2-5

[17] Fukunaga A , Miyamoto M , Cho Y , *et al* CD8+ tumor‐infiltrating lymphocytes together with CD4+ tumor‐infiltrating lymphocytes and dendritic cells improve the prognosis of patients with pancreatic adenocarcinoma. Pancreas 2004; 28: e26–e31. 14707745 10.1097/00006676-200401000-00023

[18] Mahmoud SM , Paish EC , Powe DG , *et al* Tumor‐infiltrating CD8+ lymphocytes predict clinical outcome in breast cancer. J Clin Oncol 2011; 29: 1949–1955. 21483002 10.1200/JCO.2010.30.5037

[19] Ropponen KM , Eskelinen MJ , Lipponen PK , *et al* Prognostic value of tumour‐infiltrating lymphocytes (TILs) in colorectal cancer. J Pathol 1997; 182: 318–324. 9349235 10.1002/(SICI)1096-9896(199707)182:3<318::AID-PATH862>3.0.CO;2-6

[20] Salgado R , Denkert C , Demaria S , *et al* The evaluation of tumor‐infiltrating lymphocytes (TILs) in breast cancer: recommendations by an International TILs Working Group 2014. Ann Oncol 2014; 26: 259–271. 25214542 10.1093/annonc/mdu450 PMC6267863

[21] Walker AK , Kartsonaki C , Collantes E , *et al* No additional prognostic value for MRE11 in squamous cell carcinomas of the anus treated with chemo‐radiotherapy. Br J Cancer 2017; 117: 322–325. 28641314 10.1038/bjc.2017.188 PMC5537498

[22] Anderson CM , Zhang B , Miller M , *et al* Fully automated RNAscope *in situ* hybridization assays for formalin‐fixed paraffin‐embedded cells and tissues. J Cell Biochem 2016; 117: 2201–2208. 27191821 10.1002/jcb.25606 PMC5132049

[23] Denkert C , Loibl S , Noske A , *et al* Tumor‐associated lymphocytes as an independent predictor of response to neoadjuvant chemotherapy in breast cancer. J Clin Oncol 2010; 28: 105–113. 19917869 10.1200/JCO.2009.23.7370

[24] Bankhead P , Loughrey MB , Fernández JA , *et al* QuPath: open source software for digital pathology image analysis. Sci Rep 2017; 7: 16878. 29203879 10.1038/s41598-017-17204-5 PMC5715110

[25] R Foundation for Statistical Computing . A language and environment for statistical computing. R Foundation for Statistical Computing: Vienna, Austria, 2012 https://www.R-project.org.

[26] Warrick JI , Walter V , Yamashita H , *et al* FOXA1, GATA3 and PPARγ cooperate to drive luminal subtype in bladder cancer: a molecular analysis of established human cell lines. Sci Rep 2016; 6: 38531. 27924948 10.1038/srep38531 PMC5141480

[27] De Lara S , Nyqvist J , Rönnerman EW , *et al* The prognostic relevance of FOXA1 and nestin expression in breast cancer metastases: a retrospective study of 164 cases during a 10‐year period (2004–2014). BMC Cancer 2019; 19: 187. 30819139 10.1186/s12885-019-5373-2 PMC6394077

[28] Jing X , Liang H , Hao C , *et al* Analyses of an epigenetic switch involved in the activation of pioneer factor FOXA1 leading to the prognostic value of estrogen receptor and FOXA1 co‐expression in breast cancer. Aging (Albany NY) 2019; 11: 7442–7456. 31562808 10.18632/aging.102250 PMC6782010

[29] Kollberg P , Chebil G , Eriksson P , *et al* Molecular subtypes applied to a population‐based modern cystectomy series do not predict cancer‐specific survival. Urol Oncol 2019; 37: 791–799. 31056435 10.1016/j.urolonc.2019.04.010

[30] Rouanne M , Betari R , Radulescu C , *et al* Stromal lymphocyte infiltration is associated with tumour invasion depth but is not prognostic in high‐grade T1 bladder cancer. Eur J Cancer 2019; 108: 111–119. 30654296 10.1016/j.ejca.2018.12.010

[31] van der Aa MNM , van Leenders GJLH , Steyerberg EW , *et al* A new system for substaging pT1 papillary bladder cancer: a prognostic evaluation. Hum Pathol 2005; 36: 981–986. 16153461 10.1016/j.humpath.2005.06.017

[32] Magers MJ , Lopez‐Beltran A , Montironi R , *et al* Staging of bladder cancer. Histopathology 2019; 74: 112–134. 30565300 10.1111/his.13734

[33] Vareki SM . High and low mutational burden tumors versus immunologically hot and cold tumors and response to immune checkpoint inhibitors. J Immunother Cancer 2018; 6: 157. 30587233 10.1186/s40425-018-0479-7 PMC6307306

[34] Ochoa AE , Choi W , Su X , *et al* Specific micro‐RNA expression patterns distinguish the basal and luminal subtypes of muscle‐invasive bladder cancer. Oncotarget 2016; 7: 80164. 27845906 10.18632/oncotarget.13284 PMC5348311

[35] Lin Y‐C , Chen C‐C , Cheng C‐J , *et al* Domain and functional analysis of a novel breast tumor suppressor protein, SCUBE2. J Biol Chem 2011; 286: 27039–27047. 21652720 10.1074/jbc.M111.244418 PMC3143662

[36] Hollway GE , Maule J , Gautier P , *et al* Scube2 mediates hedgehog signalling in the zebrafish embryo. Dev Biol 2006; 294: 104–118. 16626681 10.1016/j.ydbio.2006.02.032

[37] Baumgart E , Cohen MS , Neto BS , *et al* Identification and prognostic significance of an epithelial‐mesenchymal transition expression profile in human bladder tumors. Clin Cancer Res 2007; 13: 1685–1694. 17363521 10.1158/1078-0432.CCR-06-2330

[38] Liu B , Miyake H , Nishikawa M , *et al* Expression profile of epithelial‐mesenchymal transition markers in non‐muscle‐invasive urothelial carcinoma of the bladder: correlation with intravesical recurrence following transurethral resection. Urol Oncol 2015; 33: 110.e11–110.e18. 10.1016/j.urolonc.2014.08.012 25262382

[39] Yu Q , Zhang K , Wang X , *et al* Expression of transcription factors snail, slug, and twist in human bladder carcinoma. J Exp Clin Cancer Res 2010; 29: 119. 20809941 10.1186/1756-9966-29-119 PMC2942802

[40] Otto W , Breyer J , Herdegen S , *et al* WHO 1973 grade 3 and infiltrative growth pattern proved, aberrant E‐cadherin expression tends to be of predictive value for progression in a series of stage T1 high‐grade bladder cancer after organ‐sparing approach. Int Urol Nephrol 2017; 49: 431–437. 28035618 10.1007/s11255-016-1491-9

[41] Desai S , Dug Lim S , Jimenez RE , *et al* Relationship of cytokeratin 20 and CD44 protein expression with WHO/ISUP grade in pTa and pT1 papillary urothelial neoplasia. Mod Pathol 2000; 13: 1315–1323. 11144928 10.1038/modpathol.3880241

[42] Sanfrancesco J , McKenney JK , Leivo MZ , *et al* Sarcomatoid urothelial carcinoma of the bladder: analysis of 28 cases with emphasis on clinicopathologic features and markers of epithelial‐to‐mesenchymal transition. Arch Pathol Lab Med 2016; 140: 543–551. 27031776 10.5858/arpa.2015-0085-OA

[43] Shieh Y‐S , Lai C‐Y , Kao Y‐R , *et al* Expression of axl in lung adenocarcinoma and correlation with tumor progression. Neoplasia 2005; 7: 1058–1064. 16354588 10.1593/neo.05640 PMC1501169

[44] Vuoriluoto K , Haugen H , Kiviluoto S , *et al* Vimentin regulates EMT induction by slug and oncogenic H‐Ras and migration by governing Axl expression in breast cancer. Oncogene 2011; 30: 1436–1448. 21057535 10.1038/onc.2010.509

[45] Gjerdrum C , Tiron C , Høiby T , *et al* Axl is an essential epithelial‐to‐mesenchymal transition‐induced regulator of breast cancer metastasis and patient survival. Proc Natl Acad Sci U S A 2010; 107: 1124–1129. 20080645 10.1073/pnas.0909333107 PMC2824310

[46] Song X , Wang H , Logsdon CD , *et al* Overexpression of receptor tyrosine kinase Axl promotes tumor cell invasion and survival in pancreatic ductal adenocarcinoma. Cancer 2011; 117: 734–743. 20922806 10.1002/cncr.25483 PMC4403266

[47] Dadhania V , Zhang M , Zhang L , *et al* Meta‐analysis of the luminal and basal subtypes of bladder cancer and the identification of signature immunohistochemical markers for clinical use. EBioMedicine 2016; 12: 105–117. 27612592 10.1016/j.ebiom.2016.08.036 PMC5078592

[48] Lerner SP , McConkey DJ , Hoadley KA , *et al* Bladder cancer molecular taxonomy: summary from a consensus meeting. Bladder Cancer 2016; 2: 37–47. 27376123 10.3233/BLC-150037 PMC4927916

[49] Fabregat I , Malfettone A , Soukupova J . New insights into the crossroads between EMT and stemness in the context of cancer. J Clin Med 2016; 5: E37. 26985909 10.3390/jcm5030037 PMC4810108

