# Supplementary material for: Greater utility of molecular subtype rather than epithelial‐to‐mesenchymal transition (EMT) markers for prognosis in high‐risk non‐muscle‐invasive (HGT1) bladder cancer
Source: J Pathol Clin Res. 2020 May 6;6(4):238–51. doi: 10.1002/cjp2.167 (PMC7578305; doi:10.1002/cjp2.167)
Supplement: Supplementary file 1 — Supplementary materials and methods Figure S1. Positive and negative controls for all antibodies used in IHC Figure S2. RNAScope positive and negative controls Figure S3. H‐score scoring scales and ± examples Figure S4. Disease‐specific survival for regional RNAScope probe expression Table S1. Antibody information Table S2. RNAScope probes [file CJP2-6-238-s001.pdf]

Greater utility of molecular subtype rather than epithelial-to-mesenchymal transition (EMT) markers for prognosis in high-risk non-muscle-invasive (HGT1) bladder cancer

Ottley E *et al, J Pathol Clin Res*, DOI 10.1002/cjp2.167

Supplementary material

Reference numbers refer to the main manuscript

Supplementary materials and methods

Immunohistochemistry (IHC)

Automated IHC was conducted on the sections using a Bond-max autostainer (Leica Microsystems, GmbH, Wetzlar, Germany). Automated bake and dewaxing and antigen retrieval steps were conducted followed by a pre-primary blocking step using 10% BSA in 1 X PBS for 30 mins. Sections were incubated with primary antibodies at the dilutions and incubation times described in Supplementary material, Table S1, followed by use of a Bond Polymer Refine Detection kit (Leica Microsystems Inc., Newcastle, UK, Cat#DS9800), as per the manufacturer's instructions. Leica polymer and post-polymer were applied for 8 min followed by DAB for 10 min. Sections were counterstained with haematoxylin for 1 min and then manually dehydrated using graded ethanol and xylene and mounted using cover slips and DPX mountant (Sigma, Cat#06522). Stained slides were digitised using an Aperio ScanScope CS2 digital slide scanner (Leica Microsystems, GmbH, Wetzlar, Germany) at 400 x magnification. Positive and negative controls were included (Supplementary material, Figure S1). These were sections from commercial FFPE blocks (ProteoGenex, USA) or cell pellet sections known to be positive/negative for the desired protein. A no-primary negative control was included for all antibodies.

RNAScope in-situ hybridisation (ISH)

Probe choice: Two basal and luminal targets for the ISH probes were chosen based on a study by Ochoa *et al.* (2016) which clustered molecular subtypes with respect to miRNA targets [34]. *SCUBE2* and *FGFR3* were chosen as luminal targets and *EGFR* and *ZEB2* as basal targets.

Sample preparation and treatment: Tissue sections (6 µm) baked onto SuperFrost Plus slides (ThermoFisher) were baked again in a dry oven 1 hr prior to commencing the protocol. Sections were de-paraffinised with xylene for 2 x 5 min followed by 100% ethanol for 2 x 1 min and allowed to air-dry for 5 min at room temperature. Hydrogen peroxide (RNAScope, #322335) was added to the sections for 10 min at room temperature and placed in distilled water for 2 x 1 min. Target retrieval was conducted by placing the tissue sections into 1 x Target Retrieval Reagent (RNAScope, #322000) pre-heated to 100°C using a pressure cooker (Biocare Medical, DC2002) for 30 min. Slides were removed and placed in distilled water rinsed for 15 s and placed into 100% ethanol for 3 min. Slides were allowed to dry at room temperature. A hydrophobic barrier was drawn around each tissue section (ImmEdge pen, Vector Laboratories, #H-4000), allowed to dry, and RNAScope Protease Plus solution (RNAScope, #322331) was added for 30 min at 40°C. Slides were then placed in distilled water and washed for 1 x 1 min.

Detection: For detection the RNAScope 2.5 Detection Reagent – Red kit was used (RNAScope #322360). Initially, the hybridisation probes (Supplementary material, Table S2) were added to each tissue section and allowed to incubate for 2 hr at 40 °C. After hybridisation, slides were washed for 2 x 2 min with 1 x RNAScope wash buffer. A series of six hybridisation amplification (AMP 1-6) steps were then conducted, by placing the hybridisation amplification solution onto the tissue at 40°C for 30 min for AMP 1, 3 and 5 and 15 min for AMP 2, 4 and 6. Amplification steps 1-4 were conducted at 40°C and the remaining amplification steps were conducted at room temperature. A wash step was conducted for 2 x 2 min following each amplification incubation. Following amplification, detection substrate (RED working solution,

1:60 ratio of Fast RED-B to Fast RED-A) was added and incubated for 10 min at room temperature. The slides were then rinsed under running tap water. Slides were counterstained with aqueous haematoxylin for 30 sec and placed immediately into tap water. Slides were placed into 0.5% lithium carbonate solution for 10 dips and rinsed with tap water. Sections were dried completely for 15 min in a 60°C oven, dipped into fresh pure xylene and mounted with DPX mountant (Sigma, Cat#06522) and coverslip. Slides were dried overnight before scanning.

IHC and ISH assessment

Antibodies: Antibodies utilised are shown in supplementary material, Table S1. To assess EMT, N/E-cadherin, Axl, vimentin and slug/snail antibodies were used. N/E-cadherin and vimentin collectively represent the hallmarks of EMT [6] and were therefore chosen as core EMT markers. In addition, receptor tyrosine kinases (RTKs) have been shown to promote EMT [6]. Axl is a RTK which has been shown to be regulated by vimentin [5, 44]. Given this regulation by a core mediator of EMT and its association with EMT and metastasis [44-46], Axl was chosen for assessment. Slug and Snail are well characterised EMT transcription factors which bind to and suppress E-cadherin [6]. Hence, an antibody specific to both of these factors was chosen.

In relation to molecular subtypes, CK5/6 and GATA3 have been shown to correlate with basal and luminal subtypes respectively with 91% condordance with gene expression [47]. While the concordance of CK14 was found to be lower (89%) [47], consensus guidelines recommend the inclusion of both CK5/6 and CK14 to identify the basal subtype by IHC [48]. FOXA1 is closely related to GATA3 and is upregulated in luminal tumours, and was chosen as an additional luminal marker [26]. CD44, whilst being up-regulated in basal tumours [10], also plays a key role in stemness and EMT [49]. CK20 has been shown have an inverse correlation with CD44

[41] and also to be increased in luminal tumours and so was included as a molecular subtype marker.

Immunohistochemistry assessment: For Cytokeratins 5/6, 14 and 20, CD44, vimentin and Axl, cases were classified as negatively or positively stained based on criteria from the literature (Table 1).

For FOXA1, GATA3, E-cadherin, N-cadherin and slug/snail, staining was assessed using a semi-quantitative method. For each papillary and invasive region, up to 10 high-magnification images were acquired using ImageScope software (Aperio, Leica). Images were assessed for staining intensity on a four-point grading scale (0+ = negative, 1+ = weak, 2+ = moderate and 3+ = strong), and the percentage of cells staining positively were visually estimated. The overall semi-quantitative H-score was defined as the product of staining intensity and percentage of positive staining (intensity x percentage positive), as previously described [21]. Staining intensity scales were pre-determined for each antibody individually (as outlined in supplementary material, Figure S3). For FOXA1 and GATA3, nuclear staining was assessed, and membranous staining was assessed for E-cadherin and N-cadherin. Although the Slug/Snail antibody stains both nuclear and cytoplasmic components, only nuclear staining was assessed as increased nuclear expression of slug/snail indicates activation of EMT signalling [6].

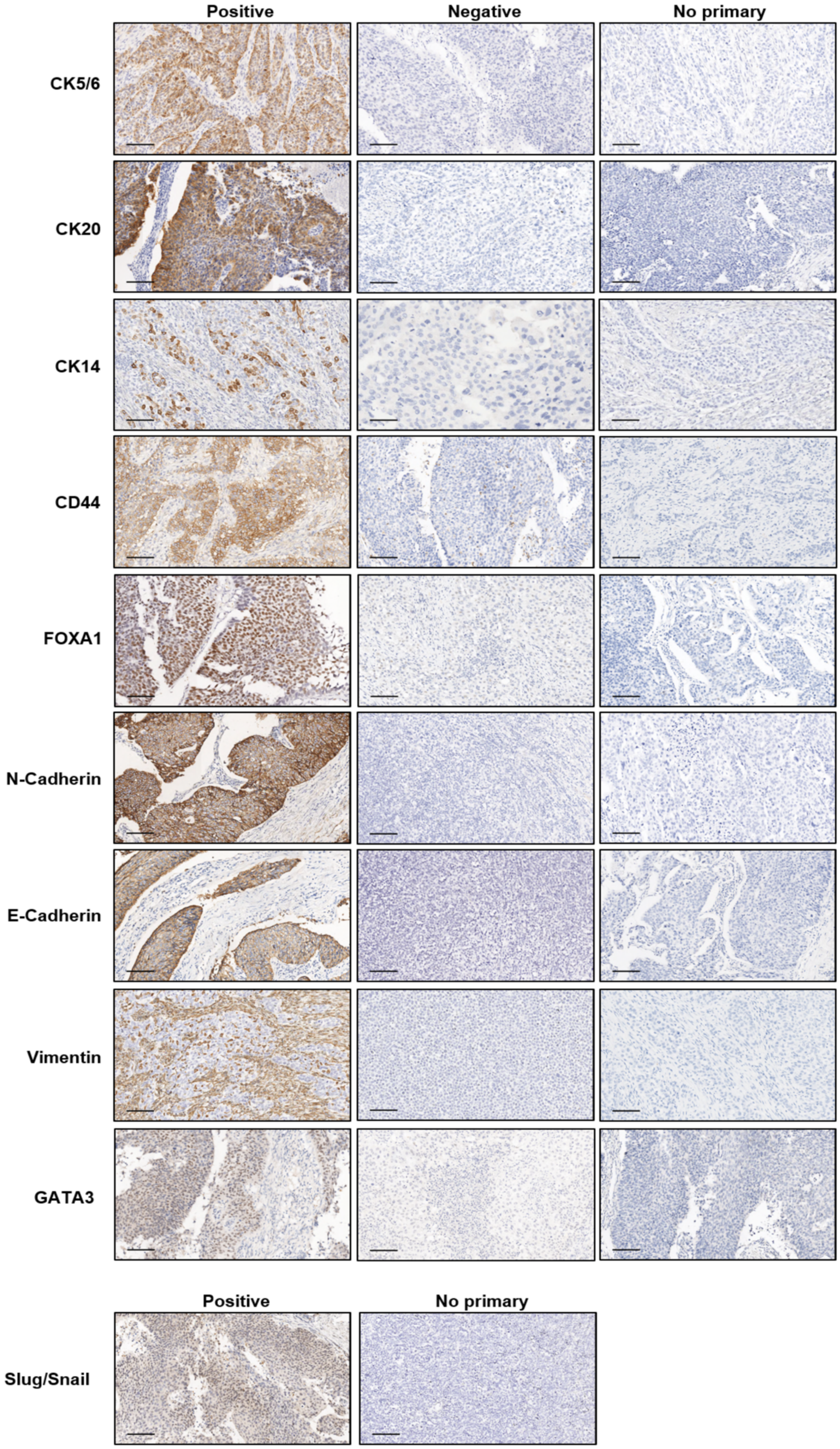
Positive
Negative
No primary
CK5/6
CK20
CK14
CD44
FOXA1
N-Cadherin
E-Cadherin
Vimentin
GATA3
Positive
No primary
Slug/Snail

Figure S1. Positive and negative controls for all antibodies used in IHC. Positive controls were sections taken from commercial blocks which were known to be positive for the protein of interest. Negative control sections were taken from commercial blocks (or cell lines) known to be negative (or reduced) for the protein of interest. For basal antibodies, negative control sections were from luminal commercial blocks where negative or low expression was expected, and vice versa for luminal antibodies. For vimentin, the negative controls were sections taken from vimentin negative CAL-29 cells. For Slug/Snail, no negative control section was available, and only a no primary negative control is shown. Scale bars = 100 µm.

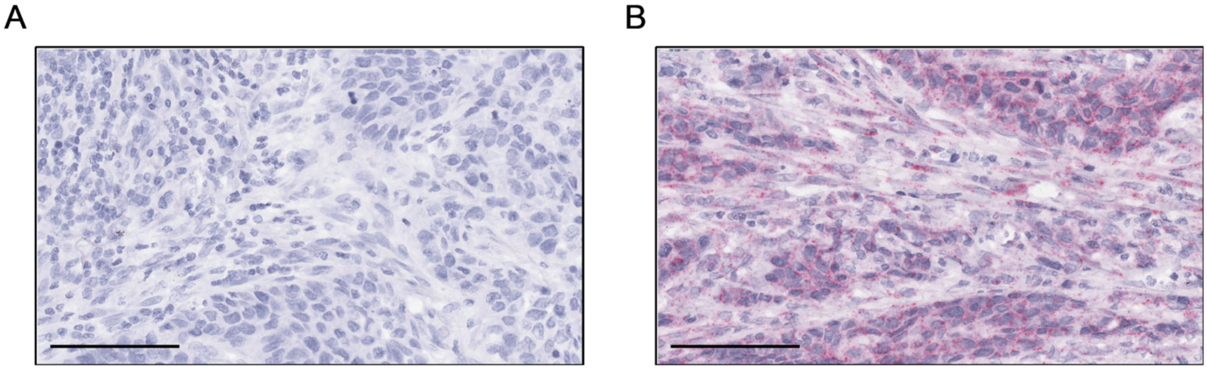

Figure S2. RNAScope positive and negative controls. Representative examples for negative control **(A)** and PPIB positive control **(B)** for the RNAScope assay. Scale bars = 100 µm.

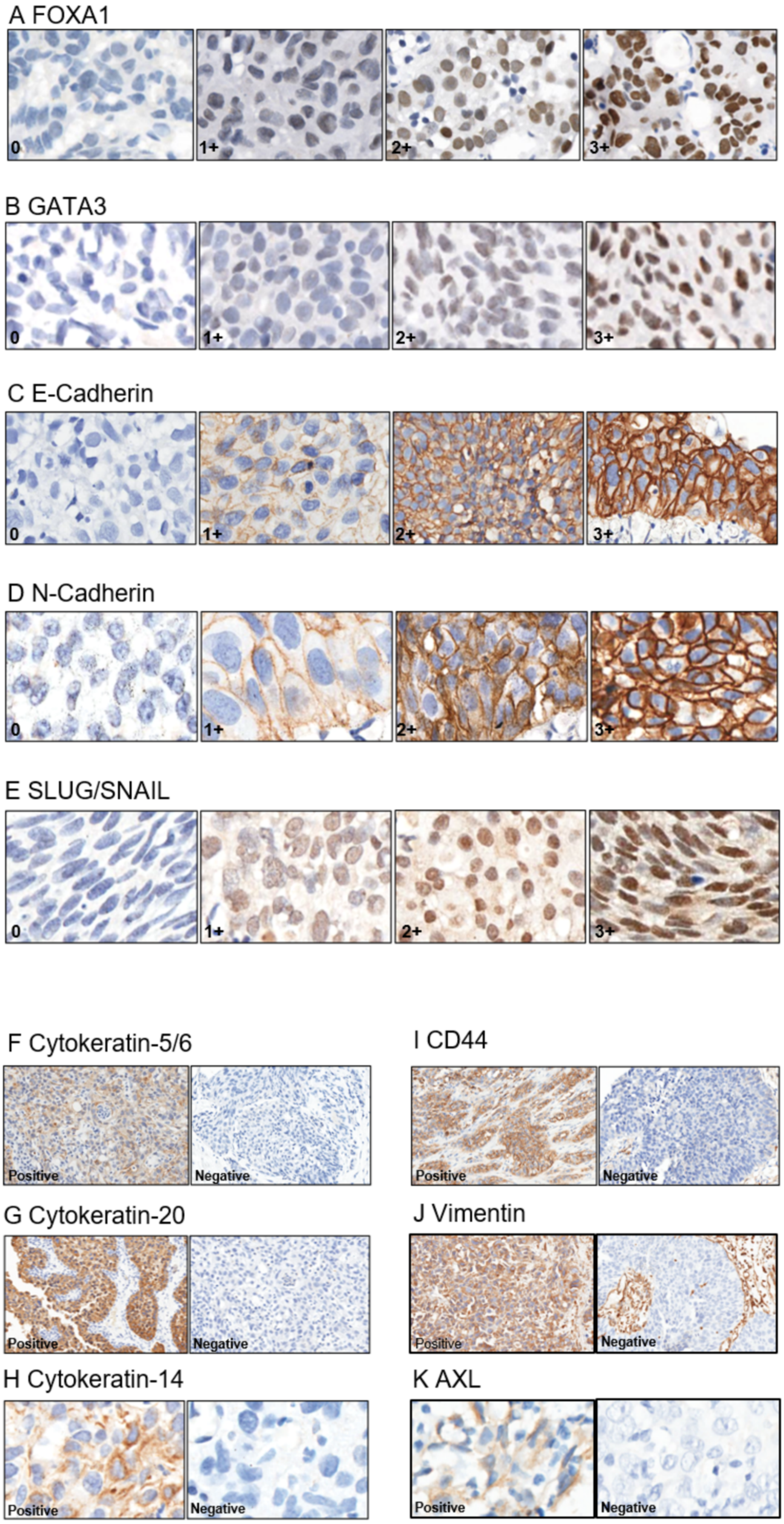
A FOXA1
0
1+
2+
3+
B GATA3
0
1+
2+
3+
C E-Cadherin
0
1+
2+
3+
D N-Cadherin
0
1+
2+
3+
E SLUG/SNAIL
0
1+
2+
3+
F Cytokeratin-5/6
Positive
Negative
I CD44
Positive
Negative
G Cytokeratin-20
Positive
Negative
J Vimentin
Positive
Negative
H Cytokeratin-14
Positive
Negative
K AXL
Positive
Negative

Figure S3. H-score scoring scales and +/- examples. Representative images for each of the staining intensities for antibodies assessed with the H-score method. The images demonstrate no staining (0), weak (1+) moderate (2+) or strong (3+) staining criteria for each antibody. **(A)** FOXA1 positive tumour nuclei (as opposed to lymphocytes) were scored**; (B)** GATA3 positive tumour nuclei were scored; **(C)** positive membranous staining was assessed for N- and **(D)** E-cadherin. **(E)** For Slug/Snail, positive tumour nuclei were scored. **(F-K)** Positive and negative criteria are also shown for the antibodies assessed with this method.

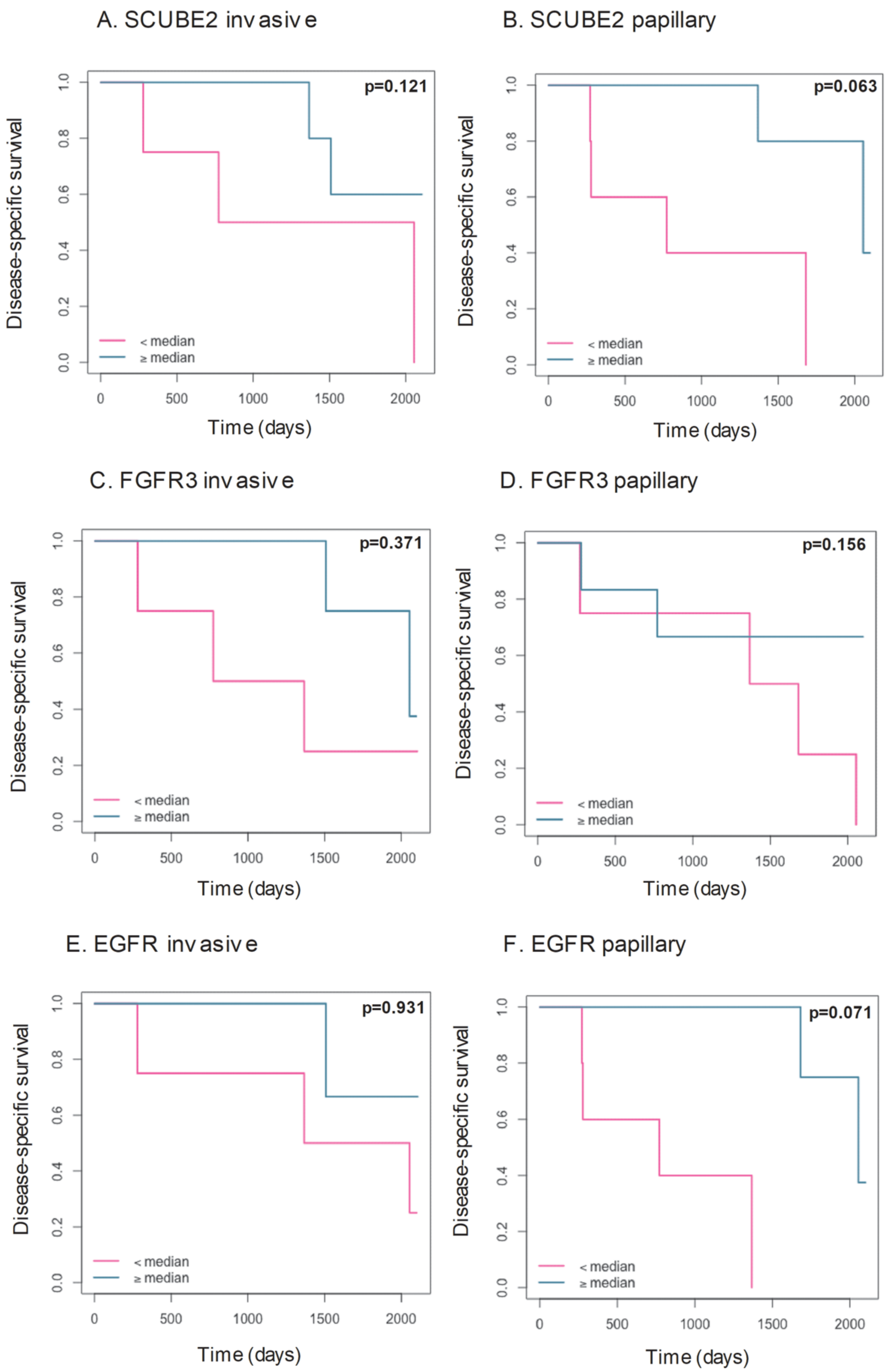

Figure S4. Disease-specific survival for regional RNAScope probe expression. Kaplan-Meier charts for disease specific survival of invasive and papillary expression for probes **(A, B)** *SCUBE2*, **(C, D)** *FGFR3* and **(E, F)** *EGFR*. The p values are stated.

Table S1. Antibody information. Antibody dilution, incubation time and source catalogue number.

Antibody	Dilution	Incubation time	Source
Basal markers			
Cytokeratin-5/6	1:500	1 hr	DAKO, M723701
Cytokeratin-14	1:1,000	1 hr	Abcam, ab7800
CD44	1:6,000	1 hr	Abcam, ab157107
Luminal markers			
Cytokeratin-20	1:3,000	1 hr	Spring Bioscience, E16444
GATA3	1:6,000	8 min	Abcam, ab199428
FOXA1	1:4,000	1 hr	Abcam, ab23738
EMT markers			
Vimentin	1:3,000	1 hr	Abcam, ab92547
E-cadherin	1:3,000	1 hr	Abcam, ab76055
N-cadherin	1:15,000	8 min	Abcam, ab98952
SLUG/SNAIL	1:3,200	1 hr	Abcam, ab85936
Axl	1:300	1 hr	Cell Signaling, 8661

Table S2. RNAScope probes. RNAScope probes used with respective catalogue reference (Advanced Cell Diagnostics, Hayward, CA, USA).

RNAScope probe name	Reference
Hs-SCUBE2	#479751
Hs-ZEB2	#404271
Hs-EGFR	#310061
Hs-FGFR3	#310791
Positive control: Hs-PPIB	#313901
Negative control: DapB	#310043
